# Isolation, identification, and genomic characterization of *Staphylococcus aureus* phage vB_SauL_202595 and its bacteriostatic application in dairy products

**DOI:** 10.1128/spectrum.01413-26

**Published:** 2026-07-29

**Authors:** Meihui Tian, Yaqian Liang, Yize Guo, Jia Lu, Yang Zhao, Hengbai Zhang, Kexun Lian, Tianyi Zhao, Hui Zhang

**Affiliations:** 1 College of Animal Science and Technology, Shihezi University 70586, Shihezi, Xinjiang, China; University of Minnesota Twin Cities, St. Paul, Minnesota, USA

**Keywords:** *Staphylococcus aureus*, phage vB_SauL_202595, class *Caudoviricetes*, biocontrol, dairy product contamination

## Abstract

*Staphylococcus aureus* is an important pathogen associated with bovine mastitis and dairy product contamination, posing economic and public health risks through the food chain. In this study, a temperate phage, vB_SauL_202595, was isolated from a dairy farm environmental sample using *S. aureus* SHZ-0127 as the host, and its biological characteristics, genomic features, and antibacterial activity in dairy matrices were evaluated. vB_SauL_202595 lysed 18 of 66 tested *S. aureus* strains, with a lysis susceptibility rate of 27.3%, including 5 highly susceptible strains, indicating a relatively limited host range. The optimal multiplicity of infection was 0.01, the latent period was approximately 30 min, and the burst size was approximately 316 PFU/cell. The phage remained stable at 4°C–37°C and pH 6–10. Genome analysis showed that vB_SauL_202595 belongs to the class *Caudoviricetes*, has a genome of 44,503 bp with 33.59% GC content, and encodes 63 predicted proteins. No typical antibiotic resistance genes or major virulence factors were detected; however, integrase and repressor genes were identified, supporting its temperate nature. vB_SauL_202595 inhibited *S. aureus* SHZ-0127 growth, reduced mature biofilm biomass, and decreased viable bacterial counts in milk and yogurt, with reductions of 1.23 and 1.42 log_10_ CFU/mL under representative conditions, respectively. From a One Health perspective, these findings provide foundational evidence for reducing *S. aureus* contamination and related antimicrobial resistance risks along the dairy chain. Overall, vB_SauL_202595 represents a candidate phage resource for dairy-associated *S. aureus* biocontrol research, but its limited host range and lysogeny-related genes require further safety assessment before food-related applications.

**IMPORTANCE:**

*Staphylococcus aureus* is a major pathogen associated with bovine mastitis and a common contaminant in dairy products, causing economic losses and public health risks through the food chain. Although phage-based biocontrol has emerged as a promising strategy for controlling *S. aureus* contamination in dairy products, systematic evidence regarding phage activity in actual dairy matrices remains limited. In this study, we isolated and characterized a dairy farm environment-derived temperate phage, vB_SauL_202595, and evaluated its biological characteristics, genomic features, host range, stability, biofilm removal ability, and antibacterial performance in milk and yogurt. These findings provide foundational experimental evidence for phage-based dairy biocontrol against *S. aureus*. However, due to its limited host range and lysogeny-related genomic features, vB_SauL_202595 should be considered a candidate phage resource for further study. Broader validation, including phage-cocktail testing, long-term storage assays, product quality assessment, and regulatory safety evaluation, is needed before practical application.

## INTRODUCTION

*Staphylococcus aureus*, belonging to the order *Staphylococcales* and the family *Staphylococcaceae*, is a key pathogen of major concern in the dairy industry (1). This widely distributed bacterium can cause bovine mastitis, leading to a sharp decline in milk yield, deterioration of milk quality, and substantial economic losses (2). The impact of *S. aureus* infection is not limited to individual animal health but also poses major challenges to dairy farmers and the entire dairy industry. Among *S. aureus* strains associated with bovine mastitis, strains carrying multidrug resistance genes and high-virulence-associated genes are frequently detected (3). In addition, this bacterium can contaminate dairy products, including raw milk and yogurt, thereby causing foodborne infections in humans. Some *S. aureus* strains carrying specific virulence-associated genes, particularly *clfB*, *hla*, and *icaA*, exhibit enhanced pathogenicity and may cause acute food poisoning, skin infections, and systemic inflammatory responses; in severe cases, they may even threaten the health of susceptible populations, such as infants, young children, and the elderly (4). Given its zoonotic transmission potential, dairy products contaminated with *S. aureus* may pose serious risks to human health (5). Recent global epidemiological studies have further demonstrated that *S. aureus* contamination is widespread and persistent throughout the dairy chain. A systematic review and meta-analysis of global milk and dairy products, including 140 studies, reported a pooled prevalence of *S. aureus* in raw milk of 33.36% (95% CI: 27.18%–39.84%), which was higher than that detected in dairy products and pasteurized milk (6). Another global meta-analysis of food contamination showed that the overall pooled prevalence of *S. aureus* in food was 30.2%, with an estimated prevalence of approximately 28.4% in dairy products (7). In addition, a 2024 review on methicillin-resistant *S. aureus* (MRSA) contamination in dairy products and bulk tank milk, which included 186 publications, found that MRSA was detected in 68.8% of dairy product-related studies and 73.8% of bulk tank milk-related studies, suggesting that the dairy production chain may serve as an important route for antimicrobial-resistant *S. aureus* to enter the food chain (8). Therefore, screening and evaluating green biocontrol resources targeting *S. aureus* from dairy production environments are of great significance for food safety and public health.

Biofilms are complex microbial communities that colonize surfaces, particularly in nutrient-rich environments, posing considerable challenges to the food industry and environmental management. As a nutrient-rich medium, milk can promote biofilm formation by *S. aureus* throughout the dairy production chain, from milking to transportation and storage (9). Biofilms present a dual challenge in antimicrobial treatment and pathogen control. On the one hand, they can reduce the efficacy of antibiotics, leading to treatment failure and increasing the risk of resistant strain emergence; on the other hand, they provide a protective barrier for pathogens, allowing them to evade immune responses and establish persistent contamination, thereby increasing the difficulty of disinfection in dairy-processing environments (10). *S. aureus*, especially multidrug-resistant strains, has the ability to produce extracellular polysaccharides and form stable biofilms. This extracellular polysaccharide-producing capacity, regulated by the *icaA* and *icaC* genes, plays an important role in the development of mature biofilms (11). In addition, studies have shown that *S. aureus* strains carrying the *cap8A* gene can produce higher levels of capsular polysaccharides and exhibit stronger biofilm-forming ability, making them more difficult to control than some other strains. Therefore, developing new approaches to effectively remove or control *S. aureus* biofilms is of great significance (12).

Bacteriophages, or phages, are naturally occurring antibacterial agents with an estimated abundance exceeding 10³¹ particles in natural environments, making them powerful tools for bacterial control (13). In the dairy industry, inhibiting the growth of pathogenic bacteria is essential for ensuring product safety and quality; therefore, phages have attracted increasing attention as a targeted biocontrol strategy (14). Previous studies have demonstrated the potential of phages to inhibit the growth of *S. aureus* in milk (15). However, the application of phages in food and dairy biocontrol still faces several limitations. First, phages generally exhibit strong host specificity, and a single phage often lyses only a subset of target strains, making it difficult to cover genetically diverse *S. aureus* populations in real dairy production environments. Second, some phages, particularly temperate phages, may carry lysogeny-associated genes, such as integrases and repressors, theoretically raising concerns about lysogenic conversion, changes in host fitness, or horizontal gene transfer. Therefore, systematic genomic safety assessment is required before food-related applications. In addition, phage efficacy in actual dairy matrices may be affected by temperature, pH, fat and protein contents, biofilm structure, and the physiological state of host cells. Thus, lytic activity observed in standard culture media may not directly reflect biocontrol performance in milk or yogurt.

From a regulatory application perspective, the use of phages as food-safety biocontrol tools has attracted international attention. Some phage preparations targeting foodborne pathogens, such as *Listeria*, *Salmonella*, and Shiga toxin-producing *Escherichia coli*, have undergone safety evaluation or received regulatory recognition for food-safety applications (16). In addition, the European Food Safety Authority has indicated that, under specific conditions, certain phages may be used for the targeted removal of specific pathogens from meat, milk, and related products (17). Nevertheless, the use of phages in food still requires case-by-case evaluation based on the specific phage strain, target bacterium, food matrix, and application conditions. Particular attention should be paid to host range, lysogeny-associated genes, the presence of antimicrobial resistance or virulence genes, application stability, and potential effects on food quality. Therefore, systematic evaluation of the biological characteristics, genomic safety, and antibacterial efficacy in actual matrices of phages originating from dairy products or dairy-related environments is an important prerequisite for advancing their application in food safety.

In this study, we systematically investigated the biological characteristics and genomic features of phage vB_SauL_202595, which was isolated using multidrug-resistant *S. aureus* SHZ-0127 as the host. We further evaluated its ability to inhibit the growth of *S. aureus* in milk and yogurt at 4°C, 25°C, and 37°C and assessed its activity against mature biofilms. By comprehensively evaluating its host range, biological properties, lysogeny-associated genomic features, genomic safety profile, and antibacterial performance in dairy matrices, this study aims to provide experimental evidence for *S. aureus* phage resource evaluation, phage-cocktail development, and the control of *S. aureus* contamination in dairy products.

## MATERIALS AND METHODS

### Bacterial strains and culture conditions

*S. aureus* SHZ-0127, maintained in our laboratory and deposited under NCBI accession number PRJNA1439548, was used as the host strain for phage isolation, purification, and propagation. Previous genomic analysis showed that this strain carries multiple virulence-associated genes, including the adhesion-related genes *clfB* and *spa*, hemolysin-related genes *hly/hla*, biofilm formation-related genes *icaA* and *icaC*, and the capsular polysaccharide synthesis-related gene *cap8A*. It also harbors several multidrug resistance-associated efflux pump genes, such as *norA*, *mepA*, *lmrS*, and *tet(38*), indicating a genetic background associated with both virulence and multidrug resistance. Before the experiments, the strain was revived and activated in Luria-Bertani (LB) broth at 37°C with shaking at 220 rpm. Bacterial cultures in the logarithmic growth phase were used for subsequent phage isolation, host range determination, optimal multiplicity of infection determination, growth inhibition assays, biofilm assays, and antibacterial assays in dairy matrices.

### Phage isolation and purification

Phage vB_SauL_202595 was isolated from an environmental wall sample collected from the milking room of a dairy farm in Yanqi County, Bayingolin Mongolian Autonomous Prefecture, Xinjiang Uygur Autonomous Region, China. Following an optimized protocol, the sample was first filtered through sterile gauze, and the filtrate was centrifuged at 6,000 rpm for 30 min. The supernatant was then sterilized by filtration through a 0.22 μm membrane. The centrifugation and filtration steps were repeated twice to remove bacteria and larger particulate impurities from the sample. Subsequently, 2 mL of the processed filtrate was mixed with 1 mL of mid-log-phase *S. aureus* SHZ-0127 culture and an appropriate volume of LB broth, followed by overnight incubation at 37°C with shaking at 220 rpm to enrich potential phages. The culture was filtered through a 0.22 μm membrane to obtain the initial phage-containing filtrate.

For phage isolation, 100 μL of the initial phage filtrate was thoroughly mixed with 100 μL of mid-log-phase *S. aureus* SHZ-0127 culture, and the mixture was plated using the double-layer agar method, followed by incubation at 37°C for 6 h. A single plaque with a clear morphology and transparent margin was picked and transferred into 1 mL of 1× PBS buffer for elution. The phage was then purified five consecutive times using the double-layer agar method until plaques with consistent size and morphology were obtained. After enrichment, the purified phage titer was determined using the double-layer agar method, and the phage stock used in subsequent experiments was confirmed to have a titer of no less than 10⁸ PFU/mL. Using *S. aureus* SHZ-0127 as the host strain, an *S. aureus* phage was successfully isolated and purified, and was designated vB_SauL_202595.

### Transmission electron microscopy observation

The morphology of phage vB_SauL_202595 was observed using phosphotungstic acid negative staining. Briefly, 20 μL of purified phage suspension was dropped onto a carbon-coated copper grid and allowed to stand for 20 min to facilitate phage particle adsorption. The grid was then negatively stained with a 2% phosphotungstic acid solution (pH 6.0) for 5 min. Excess staining solution was removed, and the grid was air-dried at room temperature. Phage morphology was observed and photographed using a Hitachi HT7800 transmission electron microscope (Hitachi, Japan) at an accelerating voltage of 80 kV and a magnification of 200,000×.

### Determination of the phage lysis spectrum

The lysis spectrum of phage vB_SauL_202595 against different *S. aureus* strains was determined using the agar spot assay. A total of 66 *S. aureus* strains were tested, among which *S. aureus* SHZ-0127 was used as the propagation host strain, while USA300 and ATCC 25923 were included as laboratory reference strains. Log-phase cultures of each bacterial strain were evenly spread onto the surface of LB agar plates. After the plates had dried, 10 μL of phage suspension was spotted onto each bacterial lawn. After the phage droplets had air-dried, the plates were inverted and incubated at 37°C. Lysis phenotypes were observed after 4 and 12 h of incubation. All experiments were performed with three independent biological replicates.

The lysis susceptibility of each strain was classified based on the morphology and transparency of the lysis zone and the presence of tolerant colonies within the lysed area. Highly susceptible strains were defined as those showing a clear and transparent lysis zone after 4 h of incubation, with the lysis zone remaining completely or nearly completely transparent after 12 h and without obvious tolerant colony growth within the lysed area. Moderately susceptible strains were defined as those showing a visible lysis zone after 4 h, but with lower transparency, less clearly defined margins, reduced lysis after 12 h, or the appearance of a small number of tolerant colonies at the edge or inside the lysis zone. Non-susceptible strains were defined as those showing no visible lysis zone or plaque formation after either 4 or 12 h of incubation. The final classification was based on lysis phenotypes consistently observed across three independent replicates.

To further validate the lysis susceptibility classification obtained from the agar spot assay, the efficiency of plating (EOP) was determined for strains showing visible lysis. Briefly, phage vB_SauL_202595 was subjected to 10-fold serial dilution, mixed with log-phase cultures of each tested strain, and then plated using the double-layer agar method. After incubation at 37°C, plaque formation was observed, and phage titers were calculated. The EOP value was calculated as follows: EOP = phage titer on the tested strain/phage titer on the propagation host strain *S. aureus* SHZ-0127. EOP assays were performed with three independent replicates and were used to support the lysis susceptibility classification shown in Fig. 2.

### Determination of the optimal multiplicity of infection

The optimal multiplicity of infection (MOI) of phage vB_SauL_202595 was determined using *S. aureus* SHZ-0127 as the host strain. Briefly, 1 mL of mid-log-phase host bacterial suspension was added to 40 mL of LB broth. Phage suspension was then added at MOIs of 100, 10, 1, 0.1, and 0.01, respectively. The mixtures were incubated at 37°C with shaking at 180 rpm for 3 h. After incubation, the cultures were centrifuged at 6,000 rpm for 10 min, and the supernatants were collected and filtered through 0.22 μm membranes. Phage titers in each group were determined using the double-layer agar method. Each experiment was performed in triplicate, and the mean value was calculated.

### One-step growth curve assay

Phage vB_SauL_202595 was mixed with log-phase *S. aureus* SHZ-0127 at the optimal MOI in 20 mL of LB broth and allowed to adsorb at 37°C for 10 min. The mixture was then centrifuged at 6,000 rpm for 10 min, the supernatant was discarded, and the bacterial pellet was resuspended in 20 mL of prewarmed LB broth to remove unadsorbed free phage particles. This washing step was repeated three times. The resuspended culture was incubated at 37°C with shaking at 180 rpm. Samples were collected every 15 min from 0 to 180 min. Phage titers at each time point were determined using the double-layer agar method. The assay was performed with three independent replicates, and the one-step growth curve was plotted based on changes in phage titer over time.

### Determination of phage thermal stability, pH stability, and UV sensitivity

To determine the thermal stability of phage vB_SauL_202595, 1 mL of phage suspension with a titer of approximately 10⁸ PFU/mL was incubated at 4°C, 25°C, 37°C, 50°C, 60°C, and 70°C, respectively. Samples were collected every 20 min from 0 to 60 min, and phage titers were determined using the double-layer agar method.

To evaluate pH stability, LB broth was adjusted to pH values ranging from 1.0 to 13.0 using hydrochloric acid or sodium hydroxide. Subsequently, 900 μL of LB broth at each pH value was mixed with 100 μL of phage suspension and incubated at 37°C with shaking at 180 rpm for 1 h, after which phage titers were determined.

To assess UV sensitivity, 15 mL of phage suspension was placed in a sterile Petri dish and exposed to a 254 nm UV lamp at a distance of 20 cm. Samples were collected at 0, 10, 20, 30, 40, 50, and 60 min, and phage titers were determined using the double-layer agar method. All experiments were performed with three independent replicates.

### *In vitro* antibacterial assay

Phage vB_SauL_202595 was mixed with *S. aureus* SHZ-0127 at MOIs of 100, 10, 1, 0.1, and 0.01, respectively. A bacterial control group inoculated with the host strain, but without phage addition, was included. All groups were incubated at 37°C with shaking at 180 rpm. The optical density at 600 nm (OD_600_) was measured hourly for 12 h using an Optizen NanoQ Plus microspectrophotometer (K LAB, Korea). The *in vitro* antibacterial activity of phage vB_SauL_202595 against the host strain was evaluated based on changes in OD_600_ over time. The experiment was performed with three independent replicates.

### Removal assay of mature biofilms by phage

The ability of phage vB_SauL_202595 to remove mature biofilms formed by *S. aureus* SHZ-0127 was evaluated using the crystal violet staining method in 96-well microplates. Briefly, 180 μL of LB broth and 20 μL of *S. aureus* SHZ-0127 suspension at approximately 10⁸ CFU/mL were added to each well and statically incubated at 37°C for 24 h to allow mature biofilm formation. After incubation, the culture medium containing planktonic cells was gently removed to eliminate non-adherent cells. Except for the biofilm-positive control group, which was inoculated with the host strain without phage addition, the remaining wells were treated with phage suspensions at titers of 10⁸, 10⁷, 10⁶, 10⁵, and 10⁴ PFU/mL, respectively. The 96-well plates were then further incubated at 37°C for 24 h.

After incubation, the liquid in each well was discarded, and 1% (wt/vol) crystal violet solution was added to each well for staining for 30 min. The wells were then gently washed two to three times with sterile saline to remove unbound dye. After the microplates were air-dried, 200 μL of absolute ethanol was added to each well to dissolve the crystal violet bound to the biofilm, followed by incubation for 30 min. Subsequently, 100 μL of the dissolved solution was transferred to a new 96-well plate, and the absorbance at 595 nm (OD595) was measured using a Multiskan GO full-wavelength microplate reader (Thermo Fisher Scientific, USA). The OD595 value was used to evaluate the residual biofilm biomass. The experiment was performed with three independent replicates.

### Whole-genome sequencing and bioinformatics analysis of the phage

After genomic DNA was extracted from purified phage vB_SauL_202595, paired-end 150 bp high-throughput sequencing was performed using the Illumina NovaSeq 6000 platform. Raw sequencing data were quality-controlled and filtered using fastp v0.23.2 to remove adapter sequences, low-quality reads, and short fragments, thereby generating high-quality clean reads. The phage genome was then assembled using Unicycler v0.5.0. Read mapping was performed using BWA v0.7.17 and Samtools v1.15.1 to evaluate sequencing depth and genome coverage.

Coding sequences (CDSs) and RNA elements in the genome were predicted using Prokka v1.14.6. RepeatMasker v4.1.2-p1 and Pseudofinder v1.1.0 were used to analyze repeat sequences and pseudogene distribution, respectively. Functional annotation of predicted proteins was performed using databases such as Pfam and UniProt, with particular attention to structural proteins, DNA packaging proteins, lysis-related proteins, and lifecycle-associated proteins. To determine the lifestyle of phage vB_SauL_202595, lysogeny-associated marker genes, including integrase, repressor, and anti-repressor genes, were specifically screened. In addition, PhiSpy v4.2.21 was used to predict potential prophage or lysogenic integration elements.

Predicted protein sequences were compared against the CARD, ARDB, and VFDB databases using Diamond to detect potential antibiotic resistance genes and virulence factors. Finally, genome structural annotation, screening results for lysogeny-associated genes, PhiSpy prediction results, and antimicrobial resistance and virulence gene database comparisons were integrated to evaluate the genomic features, lifestyle type, and safety-related genetic background of phage vB_SauL_202595.

### Antibacterial activity of phage in dairy products

Pasteurized milk and yogurt used in this study were purchased from a local market in Shihezi, Xinjiang, China, and were produced by Xinjiang Huayuan Dairy Co., Ltd. To evaluate the short-term antibacterial activity of phage vB_SauL_202595 in dairy matrices, 40 mL of fresh milk or yogurt was transferred into sterile conical flasks. Subsequently, 1 mL of *S. aureus* SHZ-0127 suspension at approximately 1 × 10⁸ CFU/mL was added and thoroughly mixed, resulting in an initial contamination level of approximately 10⁶ CFU/mL. Phage vB_SauL_202595 was then added at MOIs of 100, 10, 1, 0.1, and 0.01, respectively. A bacterial control group without phage addition was included.

The samples were stored or incubated at 4°C, 25°C, and 37°C, respectively, to simulate short-term dairy product storage under refrigerated, room-temperature exposure, and relatively high-temperature conditions. At 0, 3, 6, 9, and 12 h, 1 mL of each sample was collected, serially diluted, and subjected to plate counting to determine the viable counts of *S. aureus* in the system, expressed as CFU/mL. Changes in bacterial counts were monitored over 12 h. Each group was prepared with three independent replicates. This assay was designed to evaluate the short-term antibacterial performance of vB_SauL_202595 under different dairy matrix and temperature conditions and was not extended to long-term storage beyond 12 h.

### Statistical analysis and figure preparation

Unless otherwise stated, all experiments were performed with three independent replicates. Experimental data were analyzed using descriptive statistics and are presented as the mean ± standard deviation (mean ± SD). Error bars in the figures represent the SD of three independent replicates and were used to indicate data variability among repeated experiments. WPS Office 12.1.0 was used for data organization and descriptive statistical analysis. Origin 2025 was used to calculate means and standard deviations and to generate graphs. The circular genome map was generated using the Proksee online tool, and Adobe Illustrator 2025 was used for figure assembly and format optimization. A summary of the major experimental conditions, including MOI ranges, incubation times, temperatures, pH values, and sampling time points, is provided in Table S1.

## RESULTS

### Isolation of the phage and morphological observation by transmission electron microscopy

**Fig 1:**
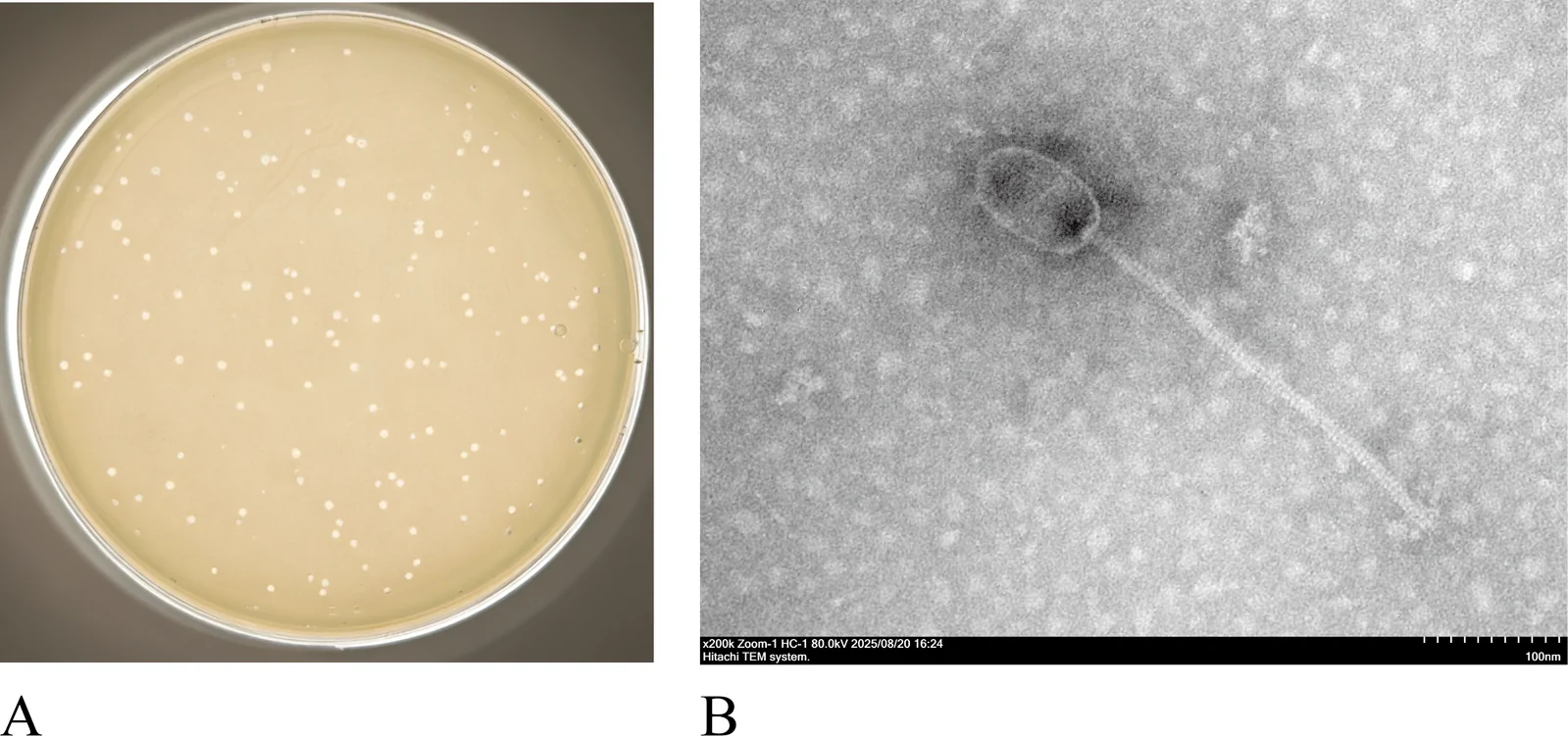
Plaque morphology and transmission electron microscopy observation of phage vB_SauL_202595. (**A**) Plaque morphology of vB_SauL_202595 formed on the bacterial lawn of *S. aureus* SHZ-0127. (**B**) Transmission electron microscopy image of vB_SauL_202595, showing an icosahedral head and a long, non-contractile tail, consistent with siphovirus-like morphological characteristics.

Using *S. aureus* SHZ-0127 as the host strain, an *S. aureus* phage was isolated and purified from a dairy farm environmental sample and designated vB_SauL_202595. After purification using the double-layer agar method, the phage formed plaques with relatively uniform morphology on the bacterial lawn of *S. aureus* SHZ-0127. The plaques were approximately 1.0 mm in diameter, transparent, and relatively regular in margin, with a faint translucent halo (Fig. 1A). These results indicated that vB_SauL_202595 could infect and lyse the host strain SHZ-0127.

Transmission electron microscopy showed that vB_SauL_202595 exhibited typical tailed phage morphology, consisting of an icosahedral head and a long, non-contractile tail, consistent with siphovirus-like morphological characteristics. According to the current ICTV taxonomy, this phage belongs to the class *Caudoviricetes*. Intact phage particles were randomly selected for measurement. The average head length and width were approximately 94.02 and 173.25 nm, respectively, while the average tail length and width were approximately 50.41 and 9.56 nm, respectively. These morphological features were generally consistent with those of some previously reported staphylococcal siphovirus-like phages (Fig. 1B).

### Determination of the phage lysis spectrum

**Fig 2:**
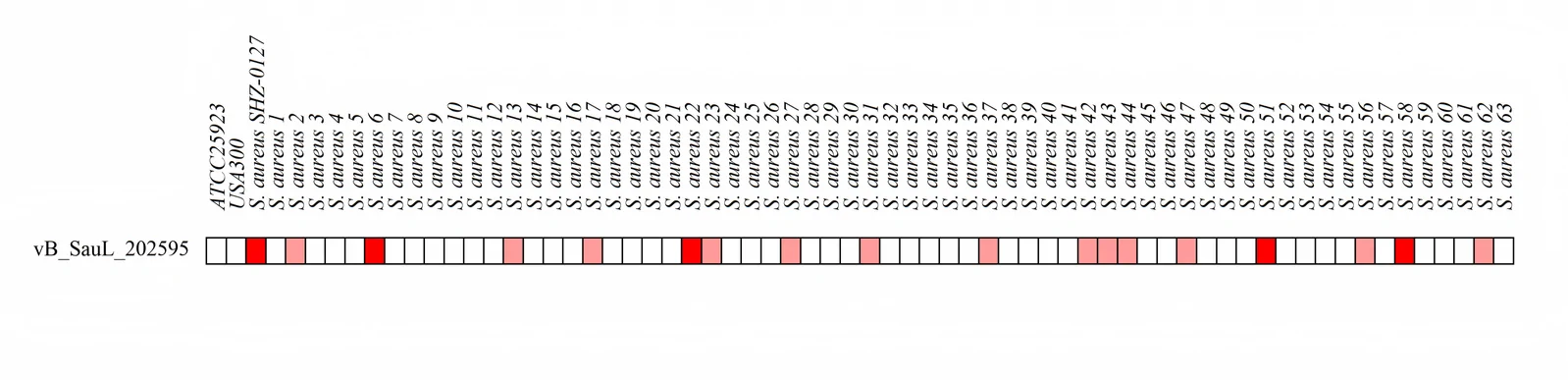
Lysis spectrum of phage vB_SauL_202595 against different *S. aureus* strains and EOP-validated susceptibility classification. A total of 66 *S. aureus* strains were tested, including the laboratory reference strains USA300 and ATCC 25923, with *S. aureus* SHZ-0127 used as the propagation host strain. Each square represents one tested strain: dark red indicates highly susceptible strains, which maintained a clear and transparent lysis zone after 12 h of incubation without obvious tolerant colony growth; light red indicates moderately susceptible strains, which showed a visible lysis zone after 4 h of incubation but exhibited tolerant colonies or weakened lysis after 12 h; white indicates non-susceptible strains, with no obvious lysis zone or plaque formation. vB_SauL_202595 lysed 18 of 66 tested *S. aureus* strains, corresponding to a lysis susceptibility rate of 27.3%, indicating a relatively limited host range. Susceptibility classification was initially determined using the agar spot assay and subsequently confirmed by EOP assays. EOP was calculated as follows: phage titer on the tested strain/phage titer on the propagation host strain SHZ-0127. All experiments were performed with three independent replicates.

The host range of phage vB_SauL_202595 was determined using the agar spot assay and further validated by the EOP assay (Fig. 2). A total of 66 *S. aureus* strains were tested for susceptibility to vB_SauL_202595, including the laboratory reference strains USA300 and ATCC 25923. The results showed that vB_SauL_202595 lysed 18 of the tested *S. aureus* strains, corresponding to a lysis susceptibility rate of 27.3% (18/66), indicating a relatively limited host range. Specifically, 5 strains were classified as highly susceptible, 13 strains as moderately susceptible, and the remaining 48 strains showed no visible lysis.

Susceptibility levels were classified based on lysis phenotypes and further confirmed by EOP assays. Strains marked in dark red were defined as highly susceptible, as they maintained a completely or nearly completely transparent lysis zone after 12 h of incubation, with no obvious tolerant colonies within the lysed area. Strains marked in light red were classified as moderately susceptible, as they showed visible lysis zones after 4 h of incubation, but tolerant colonies appeared or the lysis effect weakened after 12 h. Strains marked in white were considered non-susceptible, as no obvious lysis zone or plaque formation was observed. The EOP results were generally consistent with the classification based on the spot assay and did not change the lysis susceptibility classification shown in Fig. 2.

### Optimal multiplicity of infection and one-step growth curve

**Fig 3:**
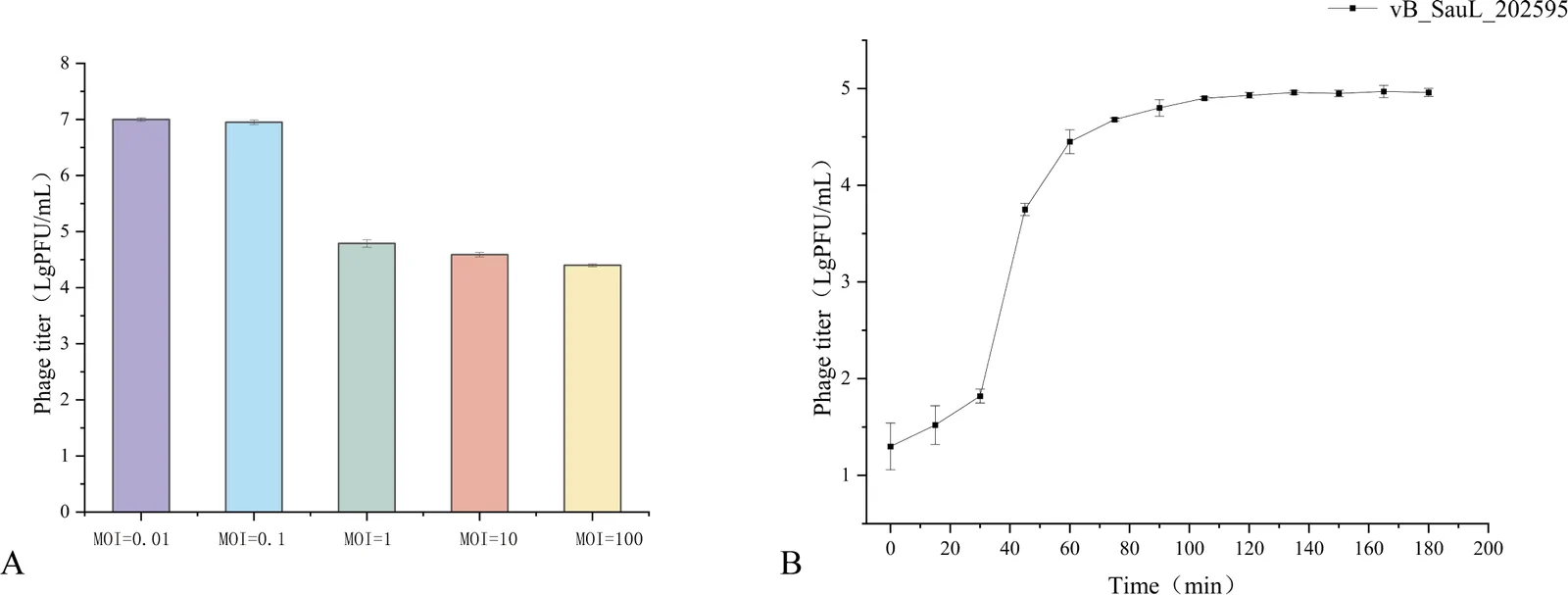
Determination of the optimal multiplicity of infection and one-step growth curve of phage vB_SauL_202595. (**A**) Propagation titers of the phage after infection of *S. aureus* SHZ-0127 at different multiplicities of infection (MOIs = 100, 10, 1, 0.1, and 0.01). The highest phage titer was observed at an MOI of 0.01, which was therefore determined as the optimal MOI for this phage. (**B**) One-step growth curve of phage vB_SauL_202595 determined under the optimal MOI condition. Samples were collected every 15 min for 180 min, and phage titers at each time point were determined using the double-layer agar method. All data were obtained from three independent replicates and are presented as the mean ± standard deviation; error bars indicate standard deviations.

The propagation titer of phage vB_SauL_202595 was determined at different multiplicities of infection (MOIs = 100, 10, 1, 0.1, and 0.01) using the double-layer agar method (Fig. 3A). The results showed that the phage reached the highest propagation titer at an MOI of 0.01, with a titer of 7.0 ± 0.04 log₁₀ PFU/mL. As the MOI increased, the phage propagation titer gradually decreased, reaching 4.4 ± 0.03 log₁₀ PFU/mL at an MOI of 100. Therefore, MOI = 0.01 was determined as the optimal MOI for vB_SauL_202595.

Using *S. aureus* SHZ-0127 as the host strain, a one-step growth curve of phage vB_SauL_202595 was generated under the optimal MOI condition (Fig. 3B). The results showed that after infection, the phage remained in the latent phase for approximately the first 30 min, during which the phage titer changed only slightly. Subsequently, the phage entered the burst phase from 30 to 90 min, during which the phage titer increased rapidly. After 90 min of incubation, the curve gradually reached a plateau, with the titer maintained at approximately 4.9 log₁₀ PFU/mL. Based on the one-step growth curve, the burst size of vB_SauL_202595 was estimated to be approximately 316 PFU/cell.

### Thermal stability, pH stability, and UV sensitivity of the phage

**Fig 4:**
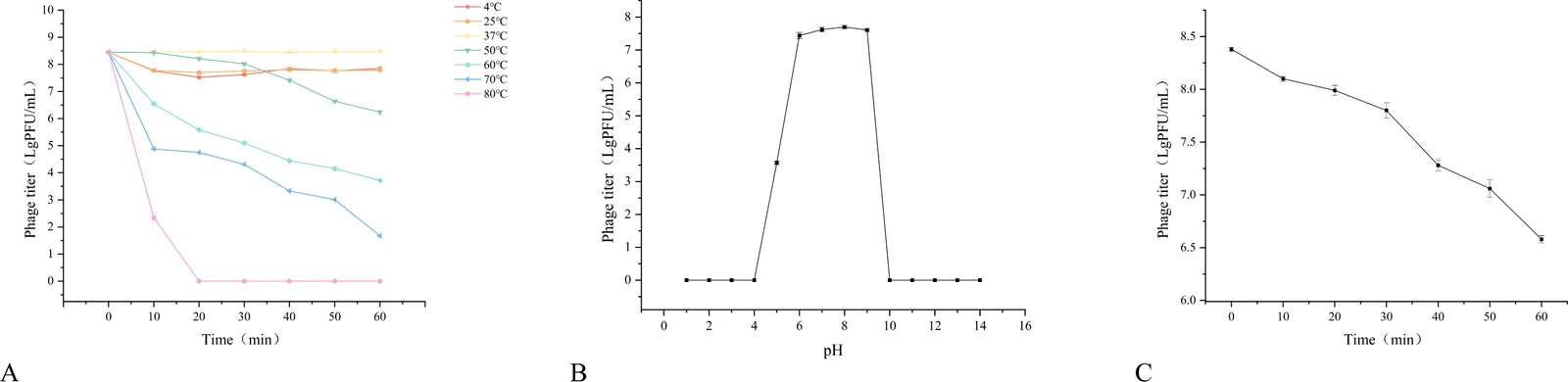
Thermal stability, pH stability, and UV sensitivity of phage vB_SauL_202595. (**A**) Changes in phage thermal stability after treatment at different temperatures for 0–60 min. (**B**) Changes in phage stability after incubation under different pH conditions. (**C**) Changes in phage titer after exposure to 254 nm UV irradiation for different durations. Phage titers were determined using the double-layer agar method and are expressed as log₁₀ PFU/mL. All data were obtained from three independent replicates and are presented as the mean ± standard deviation; error bars indicate standard deviations.

The environmental stability of phage vB_SauL_202595 is shown in Fig. 4. The thermal stability assay showed that after treatment at 4°C, 25°C, and 37°C for 60 min, the phage titers remained within 7.5–8.5 log₁₀ PFU/mL, indicating good stability under low-temperature, room-temperature, and near-conventional incubation temperature conditions (Fig. 4A). When treated at 50°C, the phage titer gradually decreased with increasing treatment time and reached approximately 6.3 log₁₀ PFU/mL at 60 min. After treatment at 60°C and 70°C, the phage titers decreased more markedly, reaching approximately 3.7 log₁₀ PFU/mL and 1.7 log₁₀ PFU/mL at 60 min, respectively, suggesting that this phage is relatively sensitive to high-temperature conditions.

The pH stability assay showed that vB_SauL_202595 maintained relatively high titers, approximately 7.5–7.8 log₁₀ PFU/mL, within the pH range of 6–10 (Fig. 4B). When the pH was lower than 5 or higher than 10, the phage titer decreased markedly, indicating that strongly acidic or alkaline conditions affected its stability. The UV sensitivity assay showed that the phage titer gradually decreased with increasing exposure time to 254 nm UV irradiation (Fig. 4C). The initial titer was approximately 8.4 log₁₀ PFU/mL and decreased to approximately 6.6 log₁₀ PFU/mL after 60 min of exposure. These results indicate that vB_SauL_202595 exhibits good stability at 4°C–37°C and pH 6–10 and has a certain tolerance to UV irradiation, but is sensitive to high temperatures and extreme pH conditions.

### *In vitro* antibacterial assay

**Fig 5:**
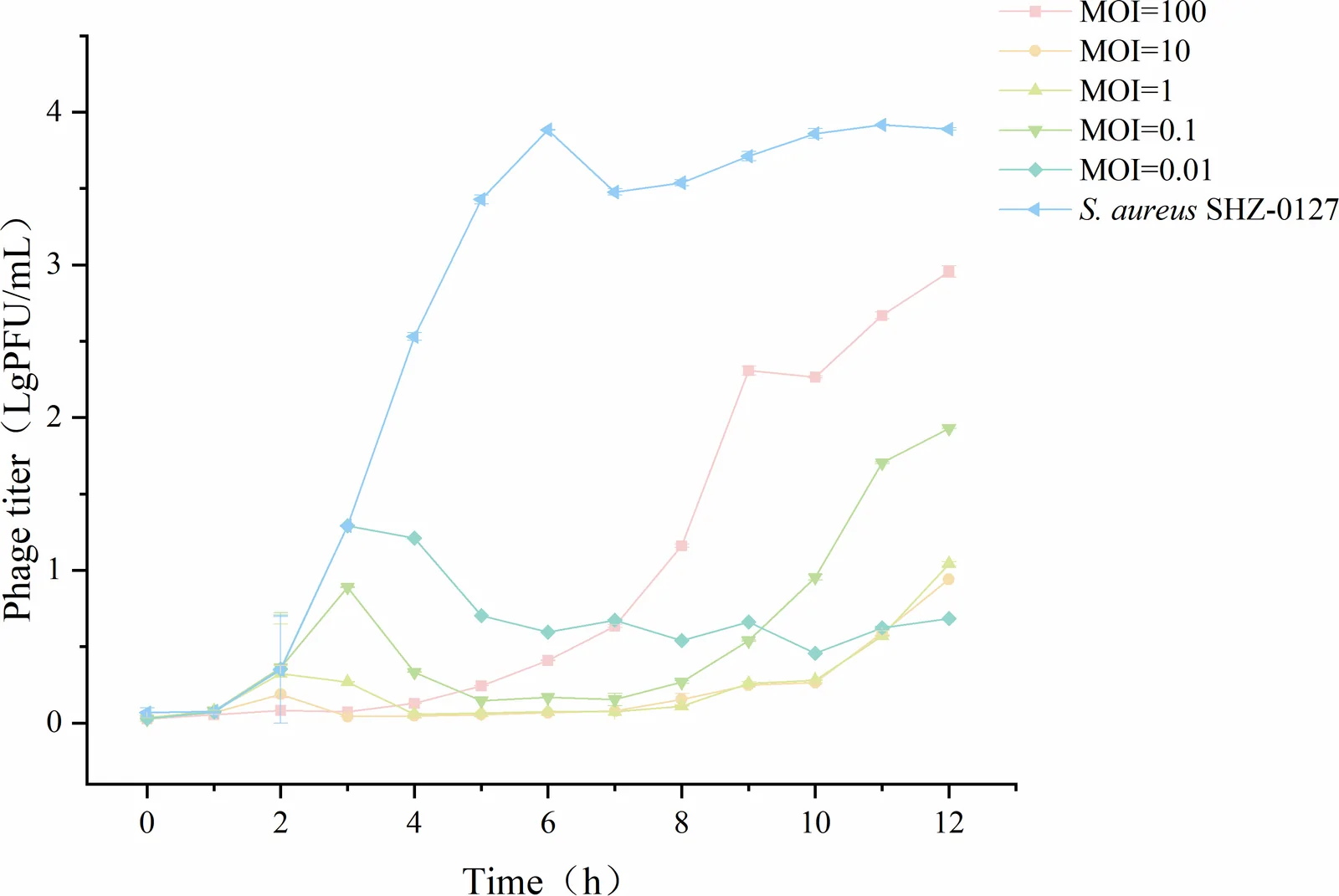
*In vitro* antibacterial activity of phage vB_SauL_202595 against *S. aureus* SHZ-0127 at different multiplicities of infection. *S. aureus* SHZ-0127 was co-cultured with phage vB_SauL_202595 at MOIs of 100, 10, 1, 0.1, and 0.01, respectively, while a bacterial control group without phage addition was included. During incubation, OD_600_ was measured every 1 h for 12 h to evaluate the inhibitory effect of the phage on host bacterial growth under different MOI conditions. The MOI = 100 group showed strong inhibition during the early incubation period, but OD_600_ increased to some extent at later time points; in contrast, the lower-MOI groups did not show a comparable late-stage recovery trend. All data were obtained from three independent replicates and are presented as the mean ± standard deviation; error bars indicate standard deviations.

To evaluate the *in vitro* antibacterial activity of phage vB_SauL_202595 against *S. aureus* SHZ-0127, phage treatment groups were established at MOIs of 100, 10, 1, 0.1, and 0.01, respectively. A bacterial control group containing only the host strain without phage addition was also included. All groups were incubated at 37°C with shaking at 180 rpm for 12 h, and OD_600_ was measured hourly to dynamically monitor bacterial growth. As shown in Fig. 5, the bacterial control group continued to grow and exhibited a typical bacterial growth pattern, whereas all phage-treated groups showed different degrees of growth inhibition during the early incubation period.

Notably, the high-MOI group (MOI = 100) showed strong inhibition of the host strain during the early stage of incubation; however, the OD_600_ value increased to some extent at later time points, suggesting that a proportion of bacterial cells may have survived under strong phage selective pressure and gradually resumed growth. In contrast, the lower-MOI groups (MOI = 0.01–1) did not show a comparable late-stage recovery trend. This may be related to the continuous amplification of phages in the host system under lower MOI conditions, gradual lysis of susceptible cells, and maintenance of a relatively stable host–phage dynamic balance. These results suggest that different MOI conditions may lead to distinct interaction patterns between the phage and the host strain. The late-stage growth recovery observed under high-MOI conditions may be associated with phage-insensitive surviving cells, escape cells, or physiological heterogeneity of the host population; however, the underlying mechanism requires further investigation.

### Phage-mediated removal of mature biofilms

**Fig 6:**
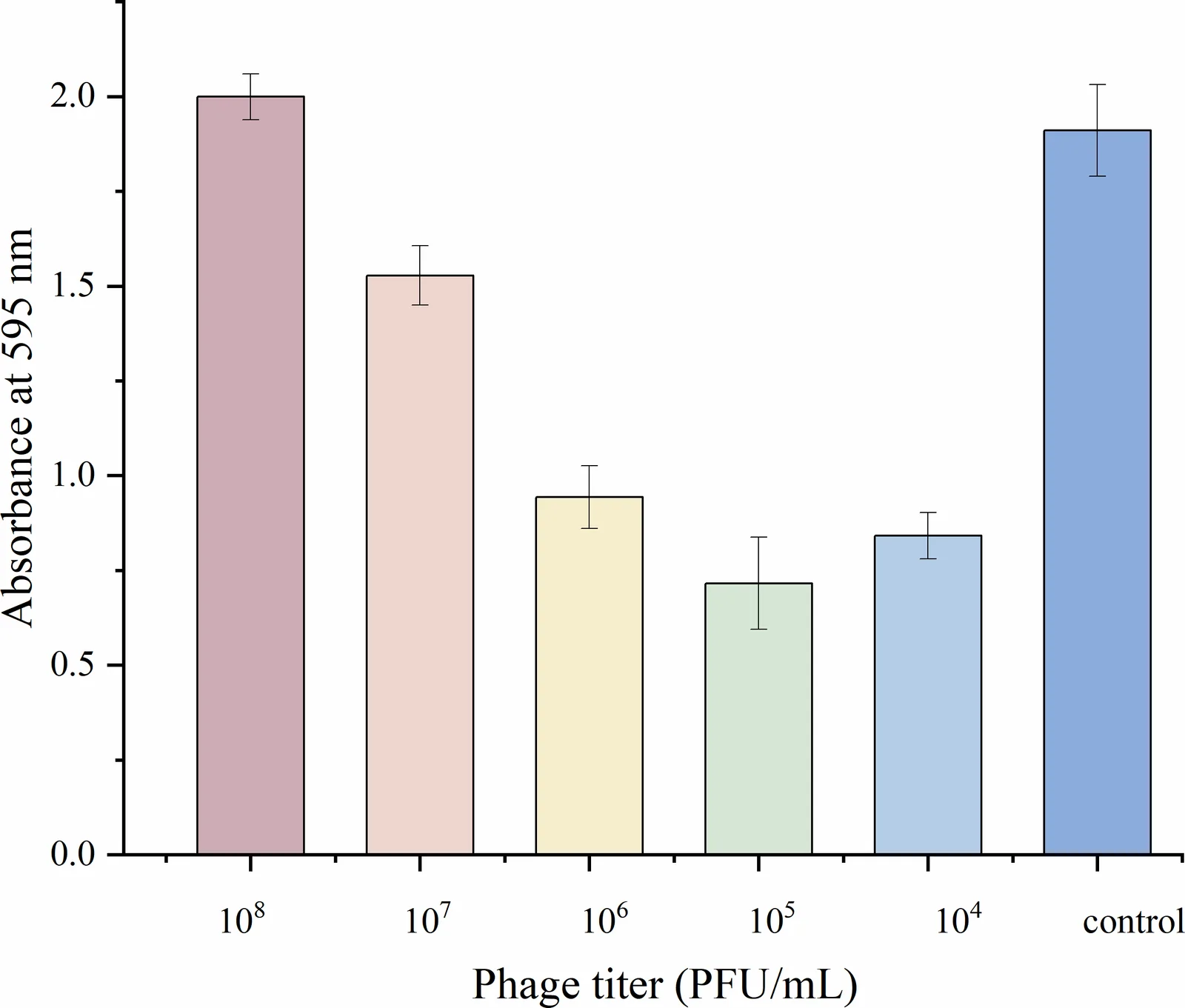
Removal effect of phage vB_SauL_202595 on mature biofilms formed by *S. aureus* SHZ-0127. The x-axis represents phage titer (PFU/mL), and the y-axis represents biofilm biomass (OD595). *S. aureus* SHZ-0127 was cultured in 96-well plates for 24 h to form mature biofilms and then treated with phage suspensions at titers of 10⁴, 10⁵, 10⁶, 10⁷, and 10⁸ PFU/mL for 24 h. The group inoculated with the host strain, but without phage addition, was used as the biofilm-positive control. Biofilm biomass was determined using the crystal violet staining method, and absorbance was measured at OD595. A lower OD595 value indicates less residual biofilm biomass. All data were obtained from three independent replicates and are presented as the mean ± standard deviation; error bars indicate standard deviations.

The removal effect of different titers of phage vB_SauL_202595 on mature biofilms formed by *S. aureus* SHZ-0127 was evaluated using the crystal violet staining method, with a biofilm-positive control group inoculated with the host strain but without phage treatment (Fig. 6). The results showed that the OD595 value of the positive control group was relatively high, indicating that the host strain was able to form mature biofilms. Compared with the control group, the OD595 values of the phage-treated groups first decreased and then increased with increasing phage titer. Within the range of 10⁴–10⁵ PFU/mL, OD595 decreased as the phage titer increased, with the 10⁵ PFU/mL group showing the lowest OD595 value and a relatively strong mature biofilm removal effect. However, when the phage titer further increased to 10⁶–10⁸ PFU/mL, OD595 gradually increased, suggesting that higher phage titers did not further enhance mature biofilm removal.

This phenomenon may be related to the inhibitory effect of mature biofilm structures on phage diffusion and adsorption. The extracellular polymeric matrix, spatial heterogeneity of cells, and differences in receptor expression within mature biofilms may reduce effective contact between high-titer phages and cells located inside the biofilm, thereby limiting phage-mediated lysis. In addition, under high phage pressure, a small number of phage-insensitive cells or escape cells may be retained and influence subsequent measurements. It should be noted that crystal violet staining mainly reflects the total residual biofilm biomass on the plate surface, including viable cells, dead cells, and extracellular matrix components. Therefore, an increase in OD595 cannot be directly interpreted as the formation of stable genetically inherited phage resistance. These results suggest that complex biofilm-protective effects or phage-insensitive phenomena may occur under high-titer phage treatment, and the underlying mechanisms require further investigation.

### Whole-genome sequencing and bioinformatics analysis

**Fig 7:**
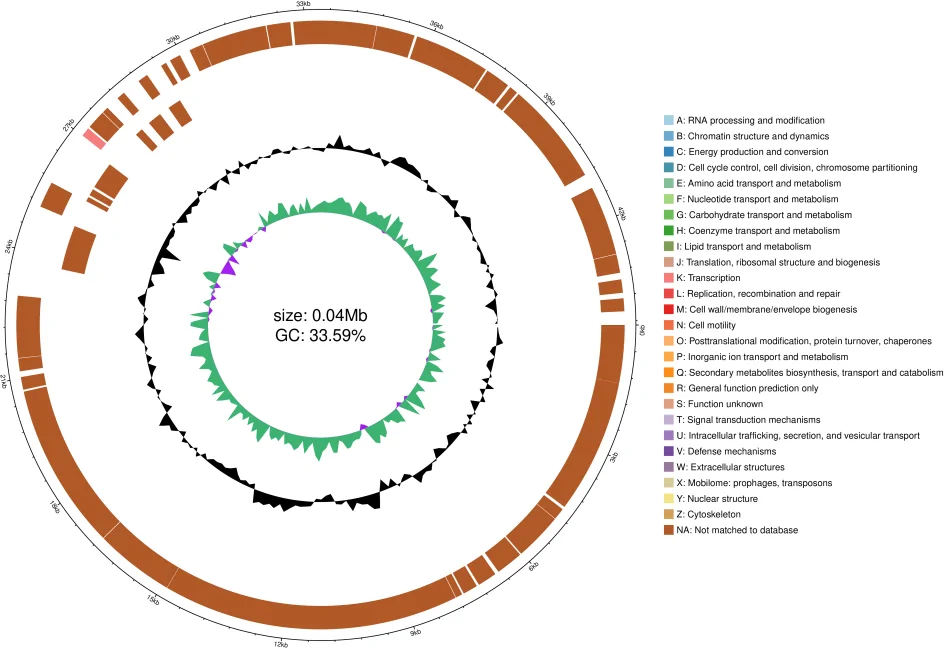
Whole-genome map and functional annotation of phage vB_SauL_202595. The figure shows the genomic structure, distribution of coding sequences, functional modules, and related annotation information of the phage genome. Genome annotation indicated that vB_SauL_202595 encodes functional elements associated with structural proteins, DNA packaging proteins, lysis-related proteins, and lysogeny-associated regulatory proteins.

Whole-genome sequencing and bioinformatics analysis were performed for phage vB_SauL_202595 (Fig. 7). After quality control and data filtering, genome assembly was completed using Unicycler, resulting in a closed circular assembled sequence. The genome of this phage was 44,503 bp in length, with a GC content of 33.59%, no sequence gaps, and an average sequencing depth of 29,224.87×, indicating high genome coverage by the sequencing data. The complete genome sequence of vB_SauL_202595 has been deposited in GenBank under accession number PZ379592. A total of 63 coding sequences (CDSs/ORFs) were predicted using Prokka (Table 1). Functional annotation showed that the genome of vB_SauL_202595 encoded multiple functional proteins associated with phage structural assembly, DNA packaging, host lysis, and lifecycle regulation. Specifically, the structural module included portal protein, capsid protein, major tail protein, tail tape measure protein, and minor structural proteins; the DNA packaging module included terminase small subunit-related proteins; and the lysis module included holin and N-acetylmuramoyl-L-alanine amidase, suggesting that this phage possesses a genetic basis associated with host cell lysis.

**TABLE 1:** Genomic characteristics and functional annotation of CDSs of phage vB_SauL_202595

CDS	Gene location	Type	Functional annotation	Homologous phage reference
1	11-1362	Phage	ORF005	PHAGE_Staphy_3A_NC_007053
2	11367-2605	Phage	Portal protein	PHAGE_Staphy_phi7401PVL_NC_020199
3	12589-3362	Phage	Prohead protease	PHAGE_Staphy_phi2958PVL_NC_011344
4	13374-4537	Phage	Putative capsid protein	PHAGE_Staphy_StauST398_2_NC_021323
5	14606-4884	Phage	Putative DNA packaging protein	PHAGE_Staphy_StauST398_2_NC_021323
6	14896-5228	Phage	Hypothetical protein	PHAGE_Staphy_Slt_NC_002661
7	15225-5626	Phage	ORF028	PHAGE_Staphy_3A_NC_007053
8	15627-6022	Phage	Hypothetical protein	PHAGE_Staphy_phi2958PVL_NC_011344
9	16057-6698	Phage	Major tail protein	PHAGE_Staphy_Slt_NC_002661
10	16790-7245	Phage	Major tail protein	PHAGE_Staphy_Slt_NC_002661
11	17303-7653	Phage	ORF034	PHAGE_Staphy_3A_NC_007053
12	17695-7853	Phage	Hypothetical protein	PHAGE_Staphy_StauST398_2_NC_021323
13	17867-14043	Phage	Phage tail tape measure protein	PHAGE_Staphy_phi7401PVL_NC_020199
14	114043-14867	Phage	Phage tail tape measure protein-like	PHAGE_Staphy_StauST398_2_NC_021323
15	114876-16459	Phage	Prophage endopeptidase tail family	PHAGE_Staphy_SMSAP5_NC_019513
16	116459-16749	Phage	Hypothetical protein	PHAGE_Staphy_StauST398_2_NC_021323
17	116765-18675	Phage	Minor structural protein	PHAGE_Staphy_StauST398_2_NC_021323
18	118675-20141	Phage	Hypothetical protein	PHAGE_Staphy_tp310_2_NC_009762
19	120141-20530	Phage	Hypothetical protein	PHAGE_Staphy_phi7401PVL_NC_020199
20	120523-20687	Phage	Hypothetical protein	PHAGE_Staphy_phi2958PVL_NC_011344
21	120721-21032	Phage	Hypothetical protein	PHAGE_Staphy_80_NC_030652
22	121168-21470	Phage	Holin	PHAGE_Staphy_phiSa2wa_st22_NC_048681
23	121481-22935	Phage	N-acetylmuramoyl-L-alanine amidase	PHAGE_Staphy_phiSa2wa_st22_NC_048681
24	123267-23566	Phage	Hypothetical protein	PHAGE_Staphy_tp310_2_NC_009762
25	123753-24958	Phage	Site-specific integrase	PHAGE_Staphy_phiSa2wa_st22_NC_048681
26	125084-25698	Phage	phi PVL ORF 30 analog	PHAGE_Staphy_phi7247PVL_NC_048624
27	125695-25820	Phage	Staphylokinase	PHAGE_Staphy_SA780ruMSSAST101_NC_048711
28	125878-26060	Phage	Hypothetical protein	PHAGE_Staphy_P282_NC_048634
29	126131-26862	Phage	Repressor	PHAGE_Staphy_tp310_2_NC_009762
30	127014-27226	Phage	Hypothetical protein	PHAGE_Staphy_tp310_2_NC_009762
31	127274-27717	Phage	Hypothetical protein	PHAGE_Staphy_tp310_2_NC_009762
32	127732-27881	Phage	Hypothetical protein	PHAGE_Staphy_tp310_2_NC_009762
33	127922-28134	Phage	Hypothetical protein	PHAGE_Staphy_tp310_2_NC_009762
34	128204-28401	Phage	Hypothetical protein	PHAGE_Staphy_tp310_2_NC_009762
35	128388-28768	Phage	ORF031	PHAGE_Staphy_3A_NC_007053
36	128835-29080	Phage	Hypothetical protein	PHAGE_Staphy_phi2958PVL_NC_011344
37	129049-29414	Phage	Hypothetical protein	PHAGE_Staphy_phi7401PVL_NC_020199
38	129469-29603	Phage	Hypothetical protein	PHAGE_Staphy_StauST398_2_NC_021323
39	129710-29973	Phage	ORF047	PHAGE_Staphy_47_NC_007054
40	130226-30549	Phage	Hypothetical protein	PHAGE_Staphy_phi7401PVL_NC_020199
41	130564-30926	Phage	ORF033	PHAGE_Staphy_3A_NC_007053
42	130923-32089	Phage	Hypothetical protein	PHAGE_Staphy_tp310_2_NC_009762
43	132115-32672	Phage	Hypothetical protein	PHAGE_Staphy_phi7401PVL_NC_020199
44	132732-34693	Phage	ORF003	PHAGE_Staphy_3A_NC_007053
45	134860-35045	Phage	Hypothetical protein	PHAGE_Staphy_phiJB_NC_028669
46	135046-35405	Phage	Hypothetical protein	PHAGE_Staphy_B236_NC_028915
47	135406-35606	Phage	phi PVL ORF 51 homolog	PHAGE_Staphy_phiPV83_NC_002486
48	135671-36063	Phage	ORF035	PHAGE_Staphy_96_NC_007057
49	136060-36251	Phage	ORF094	PHAGE_Staphy_96_NC_007057
50	136251-36682	Phage	ORF030	PHAGE_Staphy_96_NC_007057
51	136675-36923	Phage	phi PVL ORF 52 analog	PHAGE_Staphy_Slt_NC_002661
52	136916-37449	Phage	ORF020	PHAGE_Staphy_3A_NC_007053
53	137486-37731	Phage	Hypothetical protein	PHAGE_Staphy_B166_NC_028859
54	137728-37934	Phage	ORF071	PHAGE_Staphy_55_NC_007060
55	137931-38083	Phage	Integrase regulator RinB	PHAGE_Staphy_YMC/09/04/R1988_NC_022758
56	138151-38351	Phage	ORF059	PHAGE_Staphy_3A_NC_007053
57	138403-40850	Phage	ORF002	PHAGE_Staphy_3A_NC_007053
58	141191-41481	Phage	Hypothetical protein	PHAGE_Staphy_phi2958PVL_NC_011344
59	141471-42829	Phage	Helicase	PHAGE_Staphy_phi7401PVL_NC_020199
60	142842-43279	Phage	Hypothetical protein	PHAGE_Staphy_StauST398_2_NC_021323
61	143436-43750	Phage	Hypothetical protein	PHAGE_Staphy_phi2958PVL_NC_011344
62	143861-44184	Phage	Terminase-small subunit	PHAGE_Staphy_phi7401PVL_NC_020199
63	144174-44503	Phage	ORF003	PHAGE_Staphy_42E_NC_007052

During the screening of lifecycle-associated genes, site-specific integrase and repressor genes were detected in the genome of vB_SauL_202595. These genes are generally associated with the establishment and maintenance of lysogeny in temperate phages. Together with the prediction of potential lysogeny-associated elements using PhiSpy, these findings support the temperate nature of vB_SauL_202595. Therefore, this phage was classified as a temperate phage in the present study. Notably, the presence of integrase and repressor genes suggests that potential risks related to lysogenic conversion and horizontal gene transfer should be considered before food-related applications.

Homology analysis showed that most annotated ORFs in vB_SauL_202595 shared high sequence similarity with known staphylococcal phages, such as Staphy_3A, phi7401PVL, and tp310_2, supporting its taxonomic characteristics as a staphylococcal phage. Comparison with the CARD, ARDB, and VFDB databases did not identify typical antibiotic resistance genes or major virulence factors in the phage genome. This result suggests that vB_SauL_202595 does not contain clearly identifiable resistance or virulence risk genes within the scope of the databases examined. However, given its temperate genomic features, further functional validation, genetic stability analysis, and application-scenario safety assessment are still required to evaluate its feasibility for dairy biocontrol applications.

### Antibacterial activity of the phage in dairy products

**Fig 8:**
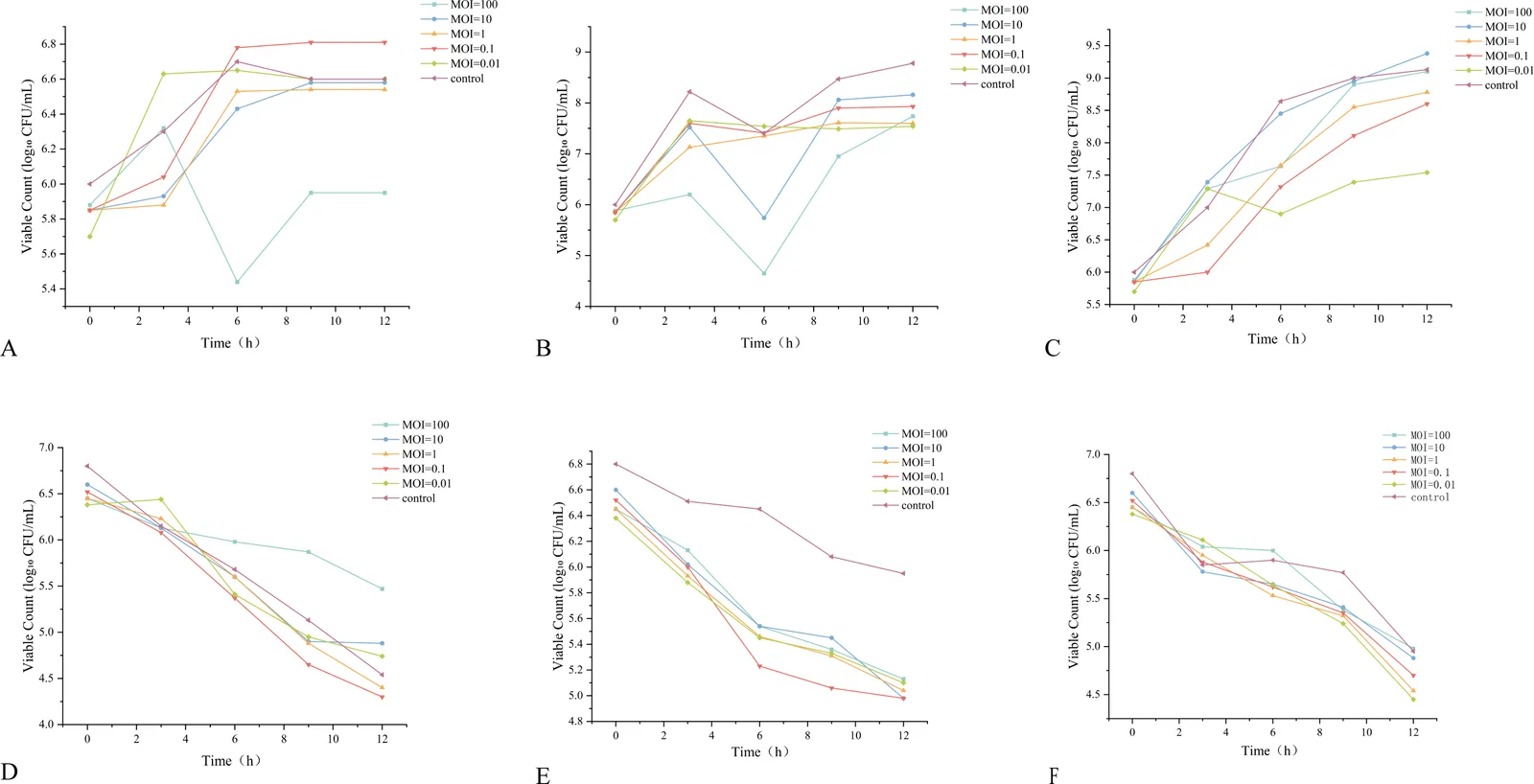
Antibacterial activity of phage vB_SauL_202595 against *S. aureus* SHZ-0127 in milk and yogurt matrices. Milk and yogurt samples were inoculated with *S. aureus* SHZ-0127 and treated with phage vB_SauL_202595 at MOIs of 100, 10, 1, 0.1, and 0.01. (A–C) Viable counts of *S. aureus* SHZ-0127 in milk at 4°C, 25°C, and 37°C, respectively; (D–F) viable counts of *S. aureus* SHZ-0127 in yogurt at 4°C, 25°C, and 37°C, respectively. Samples were collected at 0, 3, 6, 9, and 12 h, and viable counts were determined using BP selective agar and expressed as log10 CFU/mL. Data are presented as the mean ± standard deviation from three independent biological replicates; error bars indicate standard deviations.

To evaluate the antibacterial activity of phage vB_SauL_202595 in dairy matrices, milk and yogurt were used as model matrices, and changes in the viable counts of *S. aureus* SHZ-0127 were determined under different MOI treatments at 4°C, 25°C, and 37°C (Fig. 8). Viable counts were determined using Baird-Parker (BP) selective agar plates and expressed as log₁₀ CFU/mL. The antibacterial effect was expressed as the difference in viable counts between the non-phage control group and the phage-treated group under the same matrix, temperature, and time point, namely: log₁₀ reduction = log₁₀ CFU/mL of the control group − log₁₀ CFU/mL of the phage-treated group.

In the milk matrix, the viable counts of *S. aureus* SHZ-0127 in the non-phage control group increased with increasing temperature and incubation time, particularly at 25°C and 37°C. After treatment with vB_SauL_202595, different MOI groups showed varying degrees of bacterial reduction. At 4°C, host bacterial proliferation was relatively slow, and the antibacterial effect of the phage was comparatively moderate. The MOI = 100 group showed reductions of approximately 1.18 and 1.08 log₁₀ CFU/mL compared with the control group at 6 and 12 h, respectively. At 25°C, the MOI = 100 group showed a relatively pronounced early antibacterial effect, with reductions of approximately 2.45, 2.35, and 1.80 log₁₀ CFU/mL compared with the control group at 3, 6, and 9 h, respectively. At 12 h, the MOI = 0.01 group showed a reduction of approximately 0.99 log₁₀ CFU/mL compared with the control group. At 37°C, the MOI = 100 group reduced bacterial counts by approximately 2.15 log₁₀ CFU/mL compared with the control group at 3 h, but this effect weakened at later time points. In contrast, the MOI = 0.01 group showed reductions of approximately 1.48, 1.88, and 1.23 log₁₀ CFU/mL compared with the control group at 6, 9, and 12 h, respectively, indicating a more stable and sustained antibacterial trend. These results suggest that the antibacterial activity of vB_SauL_202595 in milk is clearly dependent on temperature, MOI, and incubation time.

In the yogurt matrix, the viable counts of *S. aureus* SHZ-0127 in the non-phage control group did not show the same continuous and rapid growth trend observed in milk, suggesting that the yogurt matrix itself may exert a certain inhibitory effect on bacterial proliferation. After phage treatment, viable counts decreased to varying degrees in each treatment group, although the overall antibacterial effect was weaker than that observed in milk. At 4°C, the MOI = 0.1 group showed a reduction of approximately 1.42 log₁₀ CFU/mL compared with the control group at 12 h. The MOI = 1 and MOI = 0.01 groups each showed reductions of approximately 0.72 log₁₀ CFU/mL compared with the control group at 12 h. At 25°C, the MOI = 100 group showed a reduction of approximately 0.97 log₁₀ CFU/mL compared with the control group at 3 h, while the MOI = 0.1 group showed a reduction of approximately 0.97 log₁₀ CFU/mL at 6 h. At 37°C, the low-MOI groups showed measurable log₁₀ reductions during the middle and late stages of incubation. At 9 h, the MOI = 1, 0.1, and 0.01 groups showed reductions of approximately 0.65, 0.49, and 0.34 log₁₀ CFU/mL compared with the control group, respectively. At 12 h, both the MOI = 1 and MOI = 0.01 groups showed reductions of approximately 0.37 log₁₀ CFU/mL compared with the control group.

Overall, vB_SauL_202595 reduced the viable counts of *S. aureus* SHZ-0127 to varying degrees in both milk and yogurt matrices. Compared with yogurt, the growth of the host strain was more pronounced in the milk system, and the log_10_ CFU/mL reductions produced by phage treatment were also more evident. In the milk system, the treatment group at 37°C with an MOI of 0.01 showed a reduction of approximately 1.23 log_10_ CFU/mL compared with the control group at 12 h. In the yogurt system, the treatment group at 4°C with an MOI of 0.1 showed a reduction of approximately 1.42 log₁₀ CFU/mL, compared with the control group at 12 h. These results indicate that vB_SauL_202595 has certain antibacterial potential in dairy matrices, although its efficacy is jointly influenced by matrix type, temperature, MOI, and treatment duration.

## DISCUSSION

In the dairy industry, contamination caused by multidrug-resistant and virulent *S. aureus* remains an important food safety concern, as such strains may reduce dairy product quality and increase human exposure risks through the food chain (18, 19). With increasing restrictions on conventional antimicrobial use and growing concern regarding antimicrobial resistance, targeted and environmentally friendly biocontrol strategies are urgently needed (20). Phages are promising candidates for controlling foodborne pathogens because of their host specificity and antibacterial activity; however, their application in dairy systems is still limited by narrow host range, matrix-dependent efficacy, variable lytic performance, and the need for systematic safety evaluation (21, 22). From a One Health perspective, *S. aureus* contamination in dairy products is not merely a food-processing issue, but is closely linked to bovine mastitis, milking environments, raw milk contamination, processing surfaces, environmental reservoirs, and human exposure risks. Therefore, controlling *S. aureus* in the dairy chain is relevant not only to product quality, but also to animal health, food safety, environmental management, and public health risk prevention. In this study, phage vB_SauL_202595 was isolated from a dairy farm production environment using multidrug-resistant *S. aureus* SHZ-0127 as the host strain, and its biological characteristics, genomic features, antibiofilm activity, and antibacterial performance in dairy matrices were evaluated (23).

The biological properties of vB_SauL_202595 suggest that it has a certain degree of environmental adaptability. Its optimal MOI was 0.01, indicating that the phage could propagate efficiently in the susceptible host strain at a relatively low initial input. The latent period was approximately 30 min, and the burst size was approximately 316 PFU/cell, suggesting effective replication and release in the host strain. In addition, vB_SauL_202595 remained relatively stable at 4°C–37°C and pH 6–10, covering several conditions relevant to refrigerated storage, room-temperature exposure, and short-term exposure to relatively high temperatures in dairy products (24, 25). These characteristics are partly consistent with previous reports on dairy-associated phages with low optimal MOI and good environmental stability (26–29). Nevertheless, environmental stability does not directly equate to practical food application safety. Its performance still needs to be verified under real dairy processing conditions, different storage periods, and product quality evaluation systems.

Genome analysis provided important evidence for evaluating the functional potential and safety profile of vB_SauL_202595. This phage was assigned to the class *Caudoviricetes* and exhibited siphovirus-like morphology. Its genome was 44,503 bp in length, with a GC content of 33.59%, and contained 63 predicted coding sequences. Functional annotation showed that the predicted proteins were mainly associated with structural assembly, DNA packaging, host lysis, replication and recombination, lysogeny regulation, and proteins of unknown function. Structural proteins, including capsid, head, major tail, and tail tape measure proteins, were consistent with the tailed morphology observed by transmission electron microscopy. Lysis-related proteins, including holin and N-acetylmuramoyl-L-alanine amidase, may provide a molecular basis for host cell lysis, partial biofilm removal, and reduction of viable *S. aureus* counts in dairy matrices. Similar studies on dairy-associated tailed phages have also emphasized that functional module annotation and safety screening are important foundations for evaluating phage application potential (30–33).

Importantly, vB_SauL_202595 carried lysogeny-associated genes, including site-specific integrase and repressor genes, supporting its classification as a temperate phage (31). This feature represents a key safety limitation for food biocontrol applications. Unlike strictly lytic phages, temperate phages may integrate into the bacterial genome, form lysogens, and potentially affect host fitness, virulence-associated phenotypes, biofilm-forming ability, phage susceptibility, or genetic exchange. Although no typical antibiotic resistance genes or major virulence factors were detected by screening against the CARD, ARDB, and VFDB databases, database-based analysis cannot completely exclude unknown functional genes, unannotated ORFs, lysogenic conversion effects, or low-frequency horizontal gene transfer events. Therefore, vB_SauL_202595 should not be regarded as a directly applicable single food biocontrol agent. Instead, it is more appropriately considered a candidate phage resource for mechanistic studies, lysogeny-associated safety assessment, and future phage-combination development (34). Future work should include lysogen screening, PCR- or sequencing-based verification, prophage induction assays, long-term passage stability analysis, and transduction risk assessment. Changes in host virulence, biofilm formation, antimicrobial resistance phenotype, and phage susceptibility before and after lysogenization should also be evaluated.

The host range of vB_SauL_202595 was relatively limited, which is another major factor restricting its practical application. Among 66 tested *S. aureus* strains, only 18 strains were susceptible to lysis, corresponding to a lysis susceptibility rate of 27.3%; among them, 5 strains were highly susceptible, and 13 were moderately susceptible. These results indicate that vB_SauL_202595 does not have broad-spectrum lytic activity and is unlikely to cover genetically diverse *S. aureus* populations in dairy production environments when used alone. In this study, vB_SauL_202595 was compared with several previously reported *S. aureus* phages (Table S2), but a phylogenetic tree based on whole-genome sequences or conserved core proteins was not constructed. Therefore, its phylogenetic position among known *S. aureus* phages and its evolutionary relationship with related phages remain insufficiently clarified. Future studies should further collect representative *S. aureus* phage genomes and combine whole-genome average nucleotide identity analysis, core protein clustering, and phylogenetic analysis based on conserved proteins, such as terminase and major capsid protein, to more accurately determine its taxonomic status and evolutionary background. To overcome host range limitations, strictly lytic phages with complementary host ranges may be selected to construct phage cocktails. Engineering strategies targeting receptor-binding proteins, tail fiber proteins, or tail spike proteins may also help expand host recognition, but their genetic stability, non-target effects, horizontal gene transfer risks, and regulatory acceptability must be carefully evaluated before food-related use.

The antibacterial activity of vB_SauL_202595 in dairy matrices further showed that food matrix, temperature, MOI, and treatment duration jointly influence phage efficacy. Compared with standard culture media, dairy products contain fat, proteins, acidity, and complex physical structures that may affect phage diffusion, adsorption, and contact with host cells (35, 36). In this study, vB_SauL_202595 reduced viable counts of *S. aureus* SHZ-0127 in both milk and yogurt, but the effect differed between matrices. In milk, host growth was more pronounced, and phage treatment produced more evident reductions. At 37°C, the MOI = 0.01 group reduced bacterial counts by approximately 1.23 log₁₀ CFU/mL at 12 h. In yogurt, the overall antibacterial effect was more moderate, possibly because acidity and gel structure limited phage–host interaction; however, at 4°C, the MOI = 0.1 group still reduced bacterial counts by approximately 1.42 log₁₀ CFU/mL at 12 h. These results indicate that actual food matrices, storage temperature, and host physiological status should be incorporated into the comprehensive evaluation of phage biocontrol potential. Similar findings have been reported for phage-mediated control in refrigerated milk, where dairy matrix and storage temperature were important determinants of phage efficacy (37).

In addition to reducing planktonic bacterial counts, vB_SauL_202595 showed a certain removal effect on mature biofilms formed by SHZ-0127, especially at 10⁵ PFU/mL. Biofilms are important reservoirs for persistent *S. aureus* contamination in dairy processing environments, where they can adhere to equipment, pipelines, and packaging surfaces and increase cross-contamination risk. Phages and phage-derived lysins may reduce biofilm biomass by lysing host cells, disrupting the cell wall, or affecting biofilm structure, which is consistent with previous views on the potential value of phages in whole-chain dairy contamination control (38, 39). However, higher phage titers did not further enhance mature biofilm removal. This may be associated with limited phage diffusion within the extracellular polymeric matrix, physiological heterogeneity of cells, or the presence of phage-insensitive cells. Since crystal violet staining reflects total residual biomass, including viable cells, dead cells, and extracellular matrix, increased OD₅₉₅ values under high-titer treatment cannot be directly interpreted as stable genetic phage resistance. Further studies should include residual cell isolation, EOP determination, adsorption assays, and genomic analysis to clarify the mechanism.

Several limitations should be acknowledged. First, the narrow host range limits the use of vB_SauL_202595 as a single agent in complex dairy contamination scenarios. Second, this study mainly used SHZ-0127 as the host strain and did not evaluate its efficacy in mixed bacterial communities or co-contamination models involving multiple *S. aureus* strains. Third, because vB_SauL_202595 is a temperate phage, its lysogenization capacity, horizontal gene transfer potential, genetic stability, and long-term passage safety require systematic evaluation before food-related applications. In addition, the dairy matrix assays were limited to short-term observation within 12 h and did not cover longer storage periods, cold-chain fluctuations, or temperature-abuse conditions. The effects of phage treatment on dairy product quality, including flavor, odor, color, texture, acidity, viscosity, protein stability, shelf life, fermentation characteristics, and consumer acceptance, were also not evaluated. Moreover, this study did not directly compare vB_SauL_202595 with conventional interventions, such as disinfectants, antimicrobial agents, or other food preservation technologies, under standardized conditions. Therefore, it cannot be concluded that vB_SauL_202595 is superior to existing antimicrobial or disinfection measures.

In summary, vB_SauL_202595 showed certain propagation ability in the susceptible host strain, good stability at 4°C–37°C and pH 6–10, and measurable antibacterial activity in milk and yogurt. Genome analysis did not identify typical antibiotic resistance genes or major virulence factors, but the presence of integrase and repressor genes indicates its temperate phage nature. Therefore, vB_SauL_202595 is better regarded as a candidate phage resource for research on *S. aureus* biocontrol in dairy products, rather than as a directly applicable single food biocontrol agent. Future work should focus on lysogeny-associated safety assessment, horizontal gene transfer risk, genetic stability, host phenotype changes, host range expansion, dairy product quality evaluation, and validation under practical production conditions. Strictly lytic phages with complementary host ranges, phage cocktails, engineered derivatives, or non-replicative phage-derived antibacterial components may provide safer and more feasible strategies for dairy product safety control.

### Conclusions

In this study, a temperate phage, vB_SauL_202595, was isolated and characterized using multidrug-resistant *S. aureus* SHZ-0127 carrying virulence-associated genes as the host strain. The optimal MOI of this phage was 0.01, with a latent period of approximately 30 min and a burst size of approximately 316 PFU/cell. It also maintained good stability at 4°C–37°C and pH 6–10, suggesting a certain degree of environmental adaptability. Genomic analysis showed that vB_SauL_202595 did not contain typical antibiotic resistance genes or major virulence factors; however, it carried lysogeny-associated genes, including integrase and repressor genes, supporting its temperate phage characteristics. Therefore, before food-related applications, further evaluation is still required regarding its lysogenization risk, horizontal gene transfer potential, genetic stability, and effects on host phenotypes. Dairy matrix assays showed that vB_SauL_202595 reduced the viable counts of *S. aureus* SHZ-0127 to varying degrees in milk and yogurt, and also exhibited a certain removal effect on mature biofilms. These findings provide foundational experimental evidence for the potential use of phages in controlling *S. aureus* contamination in dairy products. However, the host range of this phage was relatively limited, as it lysed only 18 of 66 tested *S. aureus* strains, among which only 5 strains were highly susceptible. This indicates that vB_SauL_202595 is not suitable for direct application as a single broad-spectrum food biocontrol agent. Overall, vB_SauL_202595 is more appropriately considered a candidate phage resource derived from dairy production environments for future studies on phage–host interactions, lysogeny-associated safety assessment, and phage cocktail development. Further studies should focus on screening phage cocktails with complementary host ranges, evaluating antibacterial stability during long-term storage and cold-chain fluctuations, assessing the sensory and physicochemical properties of dairy products, validating performance under practical production scenarios, and conducting systematic safety assessment in accordance with regulatory requirements, so as to further clarify its application feasibility in the control of *S. aureus* contamination in dairy products.

## Data Availability

The complete genome sequence of phage vB_SauL_202595 has been deposited in GenBank under accession number PZ379592. The genome data of the host strain *S. aureus* SHZ-0127 are available under NCBI BioProject accession number PRJNA1439548. All other data supporting the conclusions of this study are included in the manuscript and supplemental materials.

## SUPPLEMENTAL MATERIAL

Table S1 spectrum.01413-26-s0001.xlsx Summary of experimental conditions for phage vB_SauL_202595 assays.

Table S2 spectrum.01413-26-s0002.xlsx Comparative characteristics of vB_SauL_202595 and other reported *Staphylococcus aureus* phages.

ASM does not own the copyrights to Supplemental Material that may be linked to, or accessed through, an article. The authors have granted ASM a non-exclusive, world-wide license to publish the Supplemental Material files. Please contact the corresponding author directly for reuse.
